# Investigating Polymer–Metal Interfaces by Grazing Incidence Small-Angle X-Ray Scattering from Gradients to Real-Time Studies

**DOI:** 10.3390/nano6120239

**Published:** 2016-12-10

**Authors:** Matthias Schwartzkopf, Stephan V. Roth

**Affiliations:** 1 Deutsches Elektronen-Synchrotron (DESY), Notkestraße 85, D-22607 Hamburg, Germany; 2 KTH Royal Institute of Technology, Department of Fibre and Polymer Technology, Teknikringen 56-58, SE-100 44 Stockholm, Sweden

**Keywords:** polymer–metal interfaces, nanocomposites, nanostructure formation, sputter deposition, polymer template, X-ray scattering, grazing incidence X-ray scattering, in situ, growth kinetics

## Abstract

Tailoring the polymer–metal interface is crucial for advanced material design. Vacuum deposition methods for metal layer coating are widely used in industry and research. They allow for installing a variety of nanostructures, often making use of the selective interaction of the metal atoms with the underlying polymer thin film. The polymer thin film may eventually be nanostructured, too, in order to create a hierarchy in length scales. Grazing incidence X-ray scattering is an advanced method to characterize and investigate polymer–metal interfaces. Being non-destructive and yielding statistically relevant results, it allows for deducing the detailed polymer–metal interaction. We review the use of grazing incidence X-ray scattering to elucidate the polymer–metal interface, making use of the modern synchrotron radiation facilities, allowing for very local studies via in situ (so-called “stop-sputter”) experiments as well as studies observing the nanostructured metal nanoparticle layer growth in real time.

## 1. Introduction

In our modern information society, electronic devices based on polymer-metal-composites are increasing in relevance due to their high performance, flexibility and low-cost production. From a scientific point of view, the combination of completely different material classes opens up a vast range of different morphologies and possible applications [1,2,3,4]. The polymers are renowned for their adjustable physicochemical properties, e.g., by increasing polymer chain length or adding polar, nonpolar or conjugated functional groups to the polymer backbone, respectively, as side chain modification [5]. The efficient preparation techniques of polymer and nanoparticle thin films incorporates a wealth of methods [6]. Among them are spray-coating [7,8,9,10,11,12], spin-coating [13,14,15,16,17,18], Langmuir-Blodgett [19,20,21], dip-coating [22,23,24], printing [25,26,27], and flow stream deposition. Concerning the latter, exploiting the selective interaction of metal with polymers, Metwalli et al. investigated the growth of necklace metal nanostructures by laminar flow of gold colloidal dispersions onto block-copolymer (BCP) thin films as templates [28]. Here, one should mention the ability of spray-coating to rapidly coat and tailor three-dimensional surfaces on a large scale [29]. Further heat or solvent treatment of the installed thin films may be performed, e.g., with respect to tailoring its functional properties [30,31,32,33]. Line-type nanostructuring by nanoimprint-lithography [34] has recently been shown to be useful for ferroelectric polymeric devices consisting of one-dimensional gratings [35].

Thin films based on polymer materials offer a wealth of advanced nano- and mesostructures [14,36,37,38,39,40,41]. Moreover, the commercial availability of nanostructured polymeric materials, e.g., colloids, block-co-polymers, blends, etc., as potential templates for metal deposition emphasizes the significance for industry and advanced material science. They are thus ideal materials for tailoring nanocomposites and functional thin films. They combine in a unique way the ability for guiding nanostructuring films and maintaining their mechanical properties, e.g., their flexibility [42,43,44,45]. The combination with polymer layers leads to a desired flexibility of the full device [46] and the ability of tuning the spectral response [47]. The field of plasmonic and magnetic applications has attracted tremendous interest. This is due to the fact that block-copolymer-templates allow for metal nanopatterning with high spatial resolution [48,49]. Thus, in thin film nanocomposites, especially magnetic, optical and electrical features are added [50,51]. Al-Badri et al. [52] used designed cobalt-containing BCP to fabricate a magnetic nanocomposite material. The magnetic domains are located in the cylindrical domains. In view of ultradense magnetic data storage, Russell et al. [53] combined advanced polymer-templating using a polystyrene-block-polymethylmethacrylate (PS-*b*-PMMA) BCP with electrotemplating of magnetic nanowires. The diblock is used here as a directing component [54,55]. The use of polymer colloids opens up new possibilities for nanostructuring [56,57,58,59]. Recently, Kim et al. created hierarchical ‘raspberry-like’ three-dimensional metal-block copolymer nano-microstructures [60]. By exploiting the selective wetting of different metals, they were able to tailor the morphology from pure surface nanostructures on microspheres to hybrid microspheres. Colloidal lithography makes use of the ordered installation of polymer colloids on surfaces and subsequent deposition of metal nano- and microstructures, e.g., for metamaterial fabrication [61,62].

Metal vacuum deposition may be used for further functionalization, or creating nanocomposite metamaterials [63]. One advantage of vacuum deposition is its ability to install highly dense nanoparticle thin films with tailored morphology. Here, one may make use of the selective interaction of metals and polymer chains, making diblock co- and blend polymer films ideal for use as directing matrices [64,65,66,67,68,69] and lithographic patterning [48]. Among the methods used are evaporation [1], pulsed laser deposition [70], atomic layer deposition [71] and sputter deposition [72,73,74].

Nowadays, supported ensembles of metal nanoparticles are promising candidates in the field of applied nanotechnology and fundamental medicine due to their size-dependent, tailorable optoelectronic, electrical and catalytic properties. Plasmonic applications rely on the interaction of light with free electron gas in the confined geometry of metal nanoparticles. Here, the oscillation of the free electron leads to distinct absorption bands due to the confined geometry [75]. Putting both material classes together, a large variety of different functional morphologies on different length scales can be addressed by using diverse polymer thin films as templates for metal deposition, e.g., metal nanoparticles, nanorods and ramified nanostructures as coating or embedded in a polymer matrix. These polymer-metal-nanocomposites represent a broad spectrum of attractive applications [46] as inexpensive and flexible organic photovoltaics (OPV) [76,77,78,79,80,81], organic light emitting diodes [82,83], organic field effect transistors [84,85] and sensors [86,87,88,89,90]. Depending on their foreseen application, an efficient tailoring of the structure on different length scales ranging from micrometer to nanometer scale and below is required to fully exploit their great potential. For a targeted preparation of metal coatings with specific material properties, the utilization of sputter deposition stands out as a versatile routine method in industry and science. Despite the fact that a large variety of nanoparticles with different sizes, shapes and compositions is accessible with chemical methods, the major advantage of physical bottom-up fabrication is to reproducibly tailor the average nanoparticle size and distances with high surface coverage demanded for an efficient device performance. The early stages of metal deposition on organic surfaces are quite well understood for metal evaporation [91]. This particularly concerns factors like metal–organic interaction, interfacial chemistry, deposition rate and temperature, as well as the diffusion behavior [1,92]. However, in industrial vacuum deposition processes, sputter deposition and not evaporation is generally the method of choice [93,94]. The sputter process is much more versatile, e.g., for deposition of high-melting point metals or alloys, and provides far better adhesion. In order to manipulate and tailor nanogranular metal structures on polymer substrates using sputter deposition, fundamental investigations of the polymer–metal–interface have been the focus of research during the last two decades. Especially, grazing incidence X-ray scattering (GIXS) techniques are a powerful tool to investigate the surface, sub-surface and interface structure in soft matter, hybrid and nanocomposite thin films [7,95,96,97,98,99]. Hence, using GIXS, substantial scientific questions like how the target material, the substrate properties and process parameters affect the growth kinetics and resulting morphologies were addressed initially ex situ by sets of static samples and gradients, then in situ investigations and nowadays real-time observations during the sputter deposition process. In particular, the growth of gold nanoclusters on spin casted polystyrene thin films serves as a widespread metal-dielectric/insulator model system for exploring practical preparation conditions and functional applications of polymer-metal-nanocomposites [2,100,101,102].

This review is dedicated to presenting an overview of the possibilities of the powerful combination of sputter deposition on polymer surfaces with GIXS. A general overview over the use of GIXS for thin film characterization in general can be found, e.g., in [95,103,104,105,106]. It is thus structured as follows. In Section 2, we introduce the basic principles of GIXS (Section 2.1), of sputter deposition (Section 2.2), and provide the analysis strategies (Section 2.3) in order to establish in detail the nanostructure–function relationship. Section 3 applies these basics to the investigation of the polymer–metal interfaces. We start with the outlining of combinatorial studies (Section 3.1), where gradient samples are locally used to probe the interface structure and morphology exploiting the unique combination of GIXS and micro- and nanofocused X-ray beams. As a first step towards real-time studies, the next Section 3.2 gives an overview of the use of the so-called ‘stop-sputter technique’, where iterative sputter deposition and GIXS probing of the installed layers is used. Section 3.3 presents the real-time observations of metal layer growth on oxide and polymer surfaces. Finally, complex nanostructure formation on nanostructured polymer thin films is outlined. The review concludes with an outlook of future research directions in the field of polymer–metal interfaces.

## 2. Combining Grazing Incidence X-Ray Scattering and Sputter Deposition

Due to the much higher surface tension of metals compared to polymers, the metal atoms on surfaces are autonomously organized into small clusters in order to minimize their surface energy [64]. The optoelectronic and chemical properties of these cluster configurations, and thus their technological applications, strongly depend on the size and distance of the clusters on the surface. Accordingly, it is necessary for an efficient and controlled production of such active nanostructured surfaces by sputter deposition to understand how the growth kinetics at the polymer–metal interface affects the morphology of the cluster assemblies. Direct observations of cluster growth and mobility have been performed by real space techniques, e.g., in situ transmission electron microscopy (TEM) [107,108,109,110] and field ion microscopy (FIM) [111]. Investigating the self-organization and embedding kinetics of metals on polymers using the sputtering process was achieved ex situ with local microscopic measurement methods [39,100,102,112]. The use of non-invasive, surface sensitive techniques is therefore an essential requirement for the analysis of the growth kinetics of metal nanostructures on and in polymer surfaces. The use of X-rays has a number of advantages. X-ray methods detect a statistical average of the electron density distribution in the irradiated sample volume. In other words, the ability of X-rays to penetrate materials mediates the possibility to obtain average information, so that growth processes at the polymer–metal interface are observable.

### 2.1. GISAXS: Theoretical Background

The method of grazing incidence small-angle X-ray scattering as a versatile and surface sensitive technique for the structural characterization in reciprocal space was introduced in 1989 by J.R. Levine and J.B. Cohen [113]. This first grazing incidence small-angle X-ray scattering (GISAXS) experiment investigated the influence of the substrate temperature on growth kinetics of gold clusters deposited on glass substrates, fabricated by evaporation [114]. In order to give a brief overview of GISAXS, we introduce in this paragraph the basic ideas.

**Figure 1:**
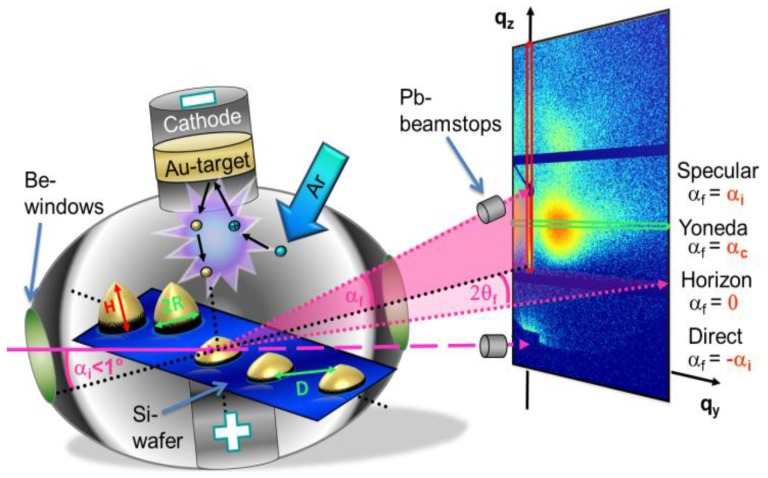
Scheme of an in situ sputter deposition experiment combined with grazing incidence small-angle X-ray scattering (GISAXS). The angle between the incident monochromatic X-ray beam and the sample surface is denoted by α*_i_*, the corresponding exit angle by α*_f_*, and the out-of-plane angle by 2θ*_f_*. A reciprocal space *(q_y_*, *q_z_)* coordinate systems is indicated. The origin of coordinates of *q_y_* and *q_z_* is indicated by the direct beam positions. The red and green rectangles in the 2D GISAXS pattern mark the region of the detector cut and out-of-plane cut, respectively. Adapted from reference [133] with permission from the Royal Society of Chemistry, 2013.

The coordinate system within the GISAXS geometry is defined such that the *x*-axis extends along the X-ray beam, the sample surface defines the *x*-*y* plane and the *z*-axis is thus oriented perpendicular to the surface, see Figure 1. The X-rays have a wavelength λ and a propagation direction along the wave vector *k_i_* within the *x*-*z* plane. They impinge on the sample at an incident angle α*_i_* with respect to the sample surface and scatter at the electron shells of the surface structures with a wave vector *k_f_*. The scattered beam reaches the detector at a vertical exit angle α*_f_* and at a horizontal angle 2θ*_f_*, indicating the scattering out of the reflection plane in the *y* direction. The elastic scattering process leads to a wave vector (momentum) transfer *q* = *k_f_* − *k_i_*. The notation *q = (q_x_*, *q_y_*, *q_z_)* is selected for its components and is defined by the framework of the scattering geometry:

(1) $$\vec{q}={\vec{k}}_{0}\left(\begin{array}{c} \cos\alpha_{f}\cos2\theta_{f}-\cos\alpha_{i}\\ \cos\alpha_{f}\sin2\theta_{f}\\ \sin\alpha_{f}+\sin\alpha_{i} \end{array}\right)$$

Specular scattering events are referred to the conditions α*_f_* = α*_i_*, 2*θ_f_* = 0, *q_x_* = *q_y_* = 0 and *q_z_* ≠ 0 (Figure 1a). The later one has the consequence that the intensity of the specular reflected beam contains specific depth information comparable to X-ray reflectometry [115,116]. In the case of non-specular diffuse scattering, lateral wave vector components *q_‖_* = (*q_x_*, *q_y_*) ≠ 0 and orthogonal wave vector components (*q*_⊥_) = (*q_z_*) ≠ 0 occur. In conventional GISAXS experiments the scattering data is mapped to a two-dimensional (2D) detector and image analysis is reduced to the components *q_z_* and *q_y_* (Figure 1). For the inherent small angles in GISAXS, one can usually assume that *q_x_* ≪ *q_y_* and *q_x_* ≪ *q_z_* regarding Equation (1). Thus the forward wave vector *q_x_* is negligible and the lateral component *q_‖_* = *q_y_* is thereby simplified, as the trigonometric functions in Equation (1) can be replaced by their first order approximations. Within this approximation, *q_y_* then becomes solely dependent on 2θ*_f_*. At α*_f_* = 0 the sample horizon is located and defines the origin of the reflection plane.

In general, the detected scattered intensity distribution *I(q_y_, q_z_)* is linked to a collective momentum transfer of an array of objects. The measured intensity is therefore directly proportional to the structure factor *S(q_y_*, *q_z_)* deduced by the interference function of the particle array and to the square of the individual particle form factor *F(q_y_*, *q_z_)*. The Fourier transform of the electron density correlation function determines the form factor *F(q_y_*, *q_z_)*, and describes the particle geometry. A detailed description of the underlying theory, including reflection and refraction effects, can be found e.g., in [104,105,116,117,118,119].

Figure 1 shows the typical 2D intensity distribution *I(q_y_*, *q_z_)* of a GISAXS pattern. The resulting distribution of the scattered intensity on the detector is visualized by a color code. The black horizontal and vertical areas result from inter-module detector gaps, where instead of the X-ray sensitive pixels the conductor paths of the readout electronics are located and thus are not part of the active area. Furthermore, cylindrical beam stops for the primary and reflected beam are recognizable, shadowing the respective beams and protecting the detector from the extremely high photon density of the primary and reflected beam.

The GISAXS measurements are carried out contactless and non-destructive, which generally promotes the use of this method for kinetical in situ studies of surface and interface processes on the nanoscale. This requires no special sample preparation, which underlines the versatility of this method. Since the method requires mapping in reciprocal space, the magnitude of the smallest detectable *q* value, and, therefore, the largest detectable size of the structures can be adjusted by changing the sample-detector distance [78,95,120,121,122]. Whereas the ultra-small scattering angles already display domain sizes and resolve larger particles, the X-ray wide-angle scattering is of great importance in the analysis of the crystal structure of nanocrystallites on surfaces. An essential advantage of GISAXS is the material selectivity by analyzing the intensity at the critical angle. When the incident or exit angle is in the order of the critical angle α*_i,f_ =* α*_c_*, a material specific feature arises, the so-called Yoneda peak [123]. Here, a the drastic variation of the Fresnel coefficients at α*_i,f_ =* α*_c_* and total reflection occur. Due to the interference of the transmitted and reflected beam, the intensity is increased and thus becomes strongly surface and interface sensitive. The material sensitivity stems from the fact that $\alpha_{c}=\sqrt{2\;Re\left(1-n\right)}\propto \sqrt{\rho }$, with *Re* being the real part of a complex number, *n* the refractive index and *ρ* the density of the material. Additional information on the composition of nanostructures can be achieved using a monochromatic X-ray beam near the absorption edge of a surface material in GISAXS measurements (anomalous grazing incidence small-angle X-ray scattering, AGISAXS) to obtain an element-specific investigation of nanostructured composites and alloys [124,125]. Kinetic studies make use of the ability to detect changes in the surface morphology as a function of time. It should be noted that energy-dependent studies necessitate longer integration times due to the necessary change in X-ray energy. With the use of X-ray transfocating optical elements in the beam path, the beam size is reduced. Due to the small incident angle, the X-ray footprint is elongated in beam direction. Thus, the local probing resolution perpendicular to the X-ray footprint can be adjusted [126,127,128,129,130,131,132]. It should be noted that the use of nanobeams in combination with GISAXS yields footprints of around 30 µm [132]. The focusing increases the photon density on the sample and thus excellent measurement statistics can be obtained. With the continuous technical improvement of storage rings for generating synchrotron radiation as a potential source of X-ray photons at the same time shortening the readout time of X-ray detectors, one is nowadays capable of realizing in situ GISAXS experiments even with very high time resolution [133]. The time-resolved structural information provides deep insight into the kinetics and mechanisms of surface processes on the nanoscale. Finally, many relevant scientific and industrial preparation and coating techniques including polymeric materials have been examined in situ in recent years, such as thermal and solvent vapor annealing of thin films [134], dip coating [135], spray coating [7,136,137,138], solution casting [139] or flow stream techniques [28,140,141,142]. The influence of appropriate process parameters such as substrate temperature, deposition rates, concentration, etc. can be analyzed directly in each case. Another decisive advantage of GISAXS measurements arises from the basic scattering geometry shown schematically in Figure 1. The geometry comprises the necessary degrees of freedom orthogonal to the beam direction and therefore allows for combining the experimental setup with diverse deposition applications for thin films and/or with complementary measuring methods such as ellipsometry, spectroscopy or atomic force microscopy (AFM) [134,139,143,144,145].

In order to quantify the structural information embedded in the two-dimensional intensity distribution, linear integration sections are performed, thus projecting the intensity profile along a component of the momentum transfer vector *q*. From the intensity distribution *I(q_y_*, *q_z_)* along the vertical section in the reflection plane, structural and morphological information perpendicular to the sample surface can be retrieved, namely in a so-called detector cut at *q_y_* = 0 nm^−1^, and a so-called off-detector cut at constant *q_y_* ≠ 0 nm^−1^, e.g., layer thicknesses, nanoparticle height distributions, and roughness. Horizontal sections along *q_y_* direction at constant *q_z_* orthogonal out from the reflection plane are adequately referred as out-of-plane cuts. Typically, this section is performed along the critical angle of the substrate material and is called Yonedacut. The horizontal intensity distribution contains a wide variety of lateral structure information of the detected objects on the substrate, such as particle geometry, lateral size distributions, center-to-center distances, spatial orientation or degree of order, i.e., the dispersity in the center-to-center distances, in the particle ensemble. To improve the measurement statistics, the intensity is integrated over the adjacent detector lines or pixels in a vertical or horizontal direction. The largest detectable lateral structural size of the sample defines the GISAXS resolution limit. This primarily depends on the sample-detector distance *D_SD_*, the pixel size of the detector and by the X-ray source and optics generated divergence of the primary beam [105,128,146,147,148,149,150]. Thus, an optimized reciprocal space resolution of the relevant nanostructures can be obtained by selecting a suitable sample-detector distance. As a brief remark, we will not go into the details of diffraction and wide-angle scattering, used to obtain information on the crystalline structure of the layers, see, e.g., [151,152,153].

### 2.2. Sputter Deposition

The basis of the vacuum coating process sputter deposition is the emission of atoms or molecules from a solid surface (target) caused by collision with noble gas cations (usually argon) with sufficiently high kinetic energy [154]. This ion bombardment is initiated by a cold plasma glow discharge from a high electric potential at a low argon partial pressure, which thus generates temporarily electrons and argon cations (Ar^+^) by further collisions. The Ar^+^ ions are accelerated by the external electromagnetic field towards the cathode and collide with the target material located there. After a cascade momentum transfer, individual atoms of the target are converted into the gas phase (sputtering). They are then deposited on a substrate facing the target from a distance (usually ~15–25 cm) in the vacuum chamber (deposition). The argon ions may be generated by the electrical potential of the direct current (DC sputtering) or by applying a radio frequency voltage field (RF sputtering). Here, the argon cations and the electrons are alternately accelerated by the oscillating field in opposite directions. The ions can no longer follow the alternating field at frequencies above 50 kHz due to their significantly lower specific charge. The oscillating motion of the electrons increases the probability of collisions with argon atoms and thus an increased ionization occurs. As a consequence, a higher sputtering rate can be attained at the same working gas pressure in comparison to DC sputtering. The significant advantage of the RF sputtering method is the additional ability to also atomize insulators, semiconductors or polymers, whereas DC sputtering is limited to conductive materials [155,156]. In both modes, an additional magnetic field in the vicinity of the target generates a Lorentz force, which deflects electrons on cycloid trajectories [157]. As a result, the number of collisions per electron increases, thus increasing the efficiency of the plasma nears the target during sputtering. This leads to a significant increase in the deposition rates for the same process pressure. On the other hand, the ions can lose a part of their kinetic energy by collisions in the gas phase, which in turn prevents a local increase in the substrate temperature. In general, the sputtered target atoms have a kinetic energy distribution up to 100 eV with a maximum around 1 to 2 eV, when they reach the substrate [93]. Thus, they expose a higher mobility on the substrate surface compared to evaporation, which plays a relevant role in forming more compact layers. In addition to the surface temperature as growth-determining process parameter, the working gas pressure also affects the surface mobility and thus the film properties [158]. The mean free path in the gas phase of the target material is increased by reducing the pressure, whereby the high kinetic energy is retained during the impact on the substrate. On the other hand, the probability of collisions of adatoms and clusters with gas atoms on the surface is lower, which also leads to higher surface mobility and less porous layers. An overview of the influence of process parameters on the surface morphology can be found, e.g., in [93].

In addition to the strong influence of the process parameters, substrate related properties influence the morphology of the sputter-deposited layer, as well. The surface roughness and local defect density promotes a preferred nucleation and homogeneous nucleation by random collision of adatoms plays a minor role [91,159]. Moreover, depending on the magnitude of the intermolecular interactions between substrate and adsorbate, three different growth modes occur. Generally, the differences in surface tensions influence growth of the second monolayer. When a Frank-van der Merwe growth mechanism is assumed, the surface tension of the adsorbate approximately matches that of the substrate and the new layers grow on an almost closed layer (layer-by-layer). In the opposite case, the Volmer-Weber growth, the intermolecular interaction of the deposited adatoms is much higher than to the substrate. Thus, no closed layer is formed and the growth proceeds in the form of clusters. The intermediate Stranski-Krastanow mode initially forms a closed wetting layer and then the island growth occurs, since the surface tension on the first monolayer is higher than on the pure substrate surface. In general, the interfacial energy between the substrate and adsorbate is compensated by the respective growth mode [160].

### 2.3. Measurement and Analysis Strategies

The dawn of third generation synchrotron sources with their available high photon flux (e.g., PETRA III, Deutsches Elektronen-Synchrotron (DESY) [161]) in combination with fast 2D single photon count detectors enabled the in situ and real-time investigation of thin film growth kinetics during deposition, ranging from sputter deposition via evaporation to solvent based methods [7,133,143,162,163,164,165,166]. The high time resolution in the millisecond regime allows the determination of kinetics of initial nucleation and subsequent cluster growth during sputter deposition and enables a precise investigation of gold cluster growth kinetics under conditions advancing towards industrial manufacturing [133,167]. This necessitates new approaches in measurement and analysis strategies.

It is of crucial importance to prevent the intense X-ray beams having an influence on the sample or growth kinetics during the experiment. In order to quantify possible X-ray beam effects during the in situ GISAXS experiments on the sample, the following measurement strategy is adopted. Prior to sputter deposition, the stability of the substrates in vacuum under irradiation by the X-ray beam is evaluated by continuously recording GISAXS pattern on the same position for a larger time period. The analysis of this temporal sequence yields the maximum allowed exposure time of the sample, so that no beam-induced changes in the GISAXS pattern can be observed. The in situ GISAXS measurements are then performed during a cyclic lateral movement of the entire sputter chamber, and thus the sample, perpendicular to the X-ray beam, so that the total exposure time is below the maximum allowed exposure time. After the metal deposition, an extended lateral GISAXS scan along the *y*-direction is performed on the sample to proof the homogeneity of the nanostructured thin film as well as to corroborate the absence of X-ray induced effects on the thin film growth [144,168,169,170].

Regarding the analysis of the obtained large data sequences, we introduced a generic model to quantify the changes in the surface morphology during metal deposition. In short, this analytical, quantitative model is based on geometrical assumptions and correlates the amount of deposited metal with the extracted temporal evolution of the key scattering features (such as maxima, see Figure 1) from the 2D reciprocal space data [171]. Structural and morphological parameters related to the growth of metallic layers such as cluster size and shape, center-to-center distance, the onset of long range connectivity and surface coverage can be deduced. This approach enabled a large variety of investigations into the influence of different process parameters (sputter rate, substrate temperature, etc.) on thin metal film morphology. The developed model concepts were confirmed with a sequence of molecular dynamics simulations (MD) based on atomistic Langevin equations and were able to reconstruct the evolution of geometrical modelled GISAXS data from Au growth on polystyrene (PS) during RF sputter deposition, considering the simplifications inherent in the geometric model [144,159]. In the case of a single metal growth on nanophase separated polymer templates, the complexity of the scattering pattern increases. Additional key scattering features like Kiessig fringes from the polymer layer and polymer domain size distribution come into play depending on either film thickness, block length ratio respectively on lamellar or cylindrical surface morphology [105,172]. In order to analyze the nanostructure and morphology of the deposited layers, model-based analysis of the GISAXS data are very useful [99,171,173]. To do so, a real space nanostructure and morphology of the layer is assumed. Subsequently, the expected GISAXS pattern is calculated, based on the scattering theory and using the local monodisperse approximation (LMA) for the coupling of structure and form factors [106], see above. In an iterative fitting routine, the parameters of the real-space model are adjusted, until agreement with the GISAXS data is achieved.

## 3. Polymer–Metal Interfaces: From Gradients to Real-Time Studies

Sputter deposition has readily and extensively been combined with in situ GIXS [106,133,170,171,174,175,176,177,178,179,180,181,182,183]. Therefore, we will review the different possibilities of investigating the polymer–metal interface with GISAXS, starting with combinatorial scanning via in situ stop-sputter experiments to real-time in situ investigations. At the same time, these investigations allow for tailoring the macroscopic optical properties of the polymer–metal interface, e.g., for antireflective coatings. Finally, the complex morphology of directional hierarchical polymer–metal nanostructures and curved templates is presented.

### 3.1. Combinatorial Investigations

**Figure 2:**
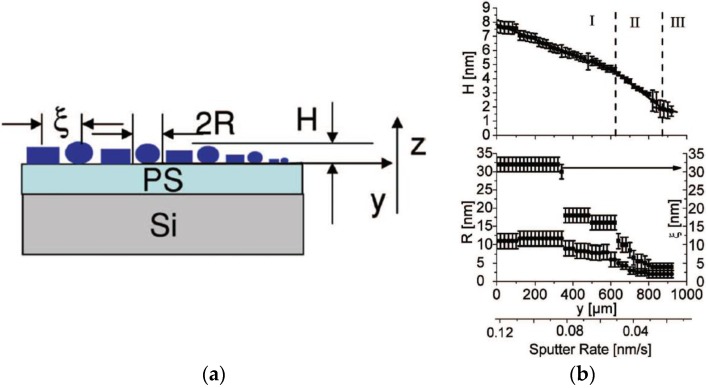
(**a**) Sketch of the nanostructure of the gradient sputter deposited Au clusters/polystyrene (PS) system. The sputter deposited Au clusters are depictured as mixture of spheroidal and cylindrical nano-objects. The gradient is in *y* direction. The clusters are on top of the PS film with height *H*, particle diameter 2*R* and center-to-center distance ξ. (**b**) Radius *R* and distance ξ as a function of position *y* in the gradient. Clearly, three regimes are visible: I–coalesced Au layer, II–isolated nanoparticle layer, III–complete suppression of coalescence, only small particles prevail. Reproduced with the permission from [150]. Copyright AIP Publishing, 2006.

For combinatorial investigations, one-dimensional (1D) gradient samples were probed using GISAXS combined with micro- and nanofocused X-ray beams [131,184,185]. Here, the 1D gradient was realized by a varying deposited Au mass thickness, leading to different nanoparticle shapes and arrangement as a function of position on the sample. This approach allowed for investigating ex situ the influence of sputter deposition [150] and evaporation rate [127] on the nanostructure. The system Au on a PS thin film with an Au gradient was investigated as a widespread metal-dielectric/insulator model system exhibiting a weak metal–polymer interaction (Figure 2a). Though optical absorption spectra of Au layers fabricated with different deposition methods show a quite similar behavior, distinct differences in the Au nanostructure at same deposited mass thickness were found. This was attributed to the different kinetic energies of evaporated atoms and sputter deposited metal ions and atoms. With the height of the nanoparticles being comparable, their in-plane structures parallel to the flat polystyrene surface were significantly larger in the case of sputter deposition [150]. The higher kinetic energy of sputtered Au atoms, when impinging on the surface, seemed to enable them to pass larger distances on the PS surface (Figure 2b). In addition, the nanoparticles' shapes and center-to-center distances were different for both coating techniques suggesting a dependence on deposition rate. The findings by GISAXS were corroborated by ex situ transmission electron microscopy (TEM), scanning electron microscopy (SEM) and atomic force microscopy (AFM). In a molecular dynamics simulation, Abraham et al. [159] have calculated the influence of the sputter rate on the structure and morphology of the metal layer. With increasing sputter rate, a decrease in distance was anticipated.

In order to increase the local probing resolution determined by the beam size, GISAXS was combined with X-ray nanobeams [129,130,186]. Depositing of conducting lines necessitates the use of shadows masks [187]. With ongoing miniaturization [188], it is thus mandatory to investigate the edge quality and extension. Therefore, firstly, Ruderer et al. used nanobeam GISAXS to determine the local gold contact morphology after sputter deposition on a photoactive diblock copolymer [189]. The extension of the tail is beyond the geometric shadow due to diffusion, and an additional selective interdiffusion of the Au in the diblock copolymer films is observed. This example nicely shows the complexity of such process technology. Secondly, imprint structures [34] are used in future memory and resistive switching devices [35]. In a pioneering experiment, a gradient Au/polymer layer morphology below the percolation threshold along a channel groove imprinted into a pressure-sensitive adhesive polymer film was studied using nanobeam GISAXS [132]. The imprinting of a macroscopically curved structure induces a gradient in the polymer thickness due to the lateral displacement of polymeric material underneath the fiber and an additional gradient in gold layer thickness as part of the surface was shadowed by the fiber during sputter deposition. This example especially shows the advantageous use of advanced GISAXS for investigating curved surfaces, when polymeric fibers are used in complex devices [190].

### 3.2. In Situ Experiments via the Stop-Sputter Technique

**Figure 3:**
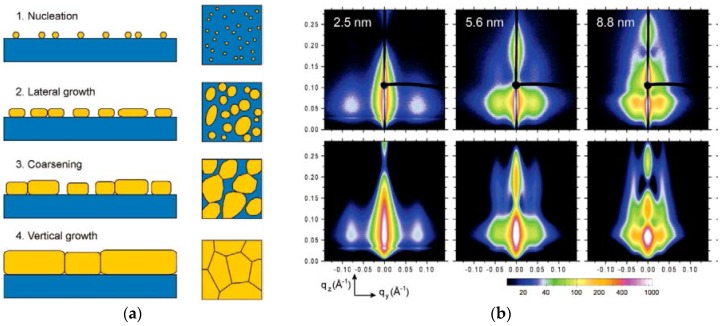
(**a**) Schematic representation of the Au cluster growth during sputter deposition on poly(*N*-vinylcarbazole) (PVK). Four growth regimes are visible with increasing surface coverage: nucleation (1), lateral cluster growth (2), coarsening (3), vertical cluster growth (4). (**b**) GISAXS pattern (top) and corresponding model-based simulations (bottom) obtained during stop-sputter deposition Au on a conducting polymer. The deposited layer thickness is indicated. Reproduced with permission from [198]. Copyright American Chemical Society, 2009.

In order to follow the metal layer build-up on and in the near-surface region, the so-called stop-sputter technique was subsequently used: After deposition of a certain metal layer thickness, the resulting nanostructure is iteratively probed by GISAXS. The investigations were extended towards more complex polymer films by including copolymer films as templates [66,67,191,192,193,194]. Using sputter deposition, Au was deposited on a blend of di- and triblock copolymers based on PS and polyisoprene (PI) [191]. The morphology of the thin blend film consisted of PS spheres in a PI matrix. Au selectively interacts with PS and thus is agglomerated on the PS spheres inside the PI matrix due to the mobility and diffusion of the Au atoms through the copolymer film [191]. Furthermore, the sputter deposition of different, technologically relevant reactive metals such as aluminum (Al), in view of electrical contacts, cobalt (Co) and iron (Fe), in view of magnetic investigations, on polymer thin films was investigated. Concerning Al, the growth of the metal layer during sputter deposition was investigated by in situ GISAXS on a semi-conducting poly(3-hexylthiophene) (P3HT) thin film. P3HT is a common material combination in organic electronic devices and organic solar cells [195,196]. As a result, it was shown that Al rapidly wets and covers the P3HT surface, leading to a homogenous, void-free film [197]. No three-dimensional cluster structures were seen due to the strong chemical interaction between Al and P3HT, which lead to a bonding of the Al atoms to the P3HT. In view of magnetic nanostructures, during sputter deposition of Co, Metwalli et al. showed that a selective decoration of PS domains occurs on microphase-separated diblock copolymer films [192]. Similarly, Schlage et al. followed the evolution of the magnetic state during Fe sputter deposition onto a highly ordered, nanoporous PS containing diblock copolymer resulting in a magnetic antidot array [193]. To gain further insight into the nucleation and growth mechanisms, Kaune et al. were able to track the gold film growth from separated clusters to a continuous film during in situ sputter deposition of an Au contact on the conducting polymer poly(9-vinylcarbazole) (PVK), spin coated on glass [198]. During the Au layer growth, four different regimes were deduced, depending on the deposited mass thickness. Although not explicitly observed, nucleation has to take place in the first regime below Au monolayer coverage. Once a critical nucleus density reached, lateral cluster growth occurs. This transition occurs, when the probability for a diffusing adatom to be captured by an existing cluster is much higher than that for it to join with a second adatom to a new nucleus [160,198]. In the third regime, coarsening of the existing clusters takes places until these formed clusters come into contact. A significant faceting is observed. Finally, when a surface coverage of 1 is reached, only vertical growth is observed. These different regimes are schematically illustrated in Figure 3. Here, one can also observe the excellent agreement between GISAXS data and model-based simulations, allowing for clearly deducing the details of layer morphology.

It should be noted, that the cluster height follows a non-linear growth law. This reflects the reduced condensation coefficient for gold particles on the PVK surface, as in general the condensation coefficients of metals on polymers are significantly lower than 1 [199]. All studies have in common, that alternating sputter deposition/measurement cycles were applied to deduce the metal layer growth, which already allowed for deducing basic governing growth laws.

### 3.3. Real-Time Observations of Metal Layer Growth

Here, we present five examples for real-time GISAXS studies during the sputter deposition process. Starting with a bimaterial model system, the sputter deposition of Au on amorphous silicon oxide (SiO*_x_*) was investigated, leading to a generic analytic growth model. This was subsequently applied to the growth of Ag on SiO*_x_* in order to elucidate the strong enhancement of the surface-enhanced Raman scattering (SERS) of such Ag nanostructures. Complexity was further increased by introducing a small-molecule layer (tris(8-hydroxyquinolinato)-aluminum, Alq3) and using Ag and Al as metal. Finally, the sputter deposition of Au on PS in combination with complementary in situ ultraviolet–visible light (UV-Vis) spectroscopy is presented.

**Figure 4:**
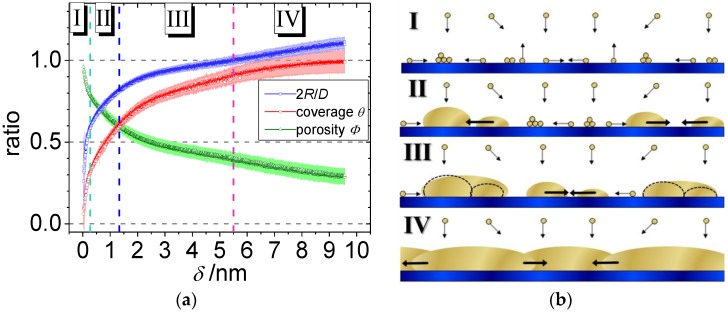
Au sputter deposition on amorphous silicon oxide: (**a**) Evolution of aspect ratio 2*R/D* (blue symbols, *R* = radius, *D* = center-to-center distance of the Au clusters), coverage θ (red symbols) and porosity Φ (green symbols) as a function of effective thickness δ ≈ 0.0032 nm/frame of Au on SiO*_x_*. (**b**) Schematic side view of the four gold cluster growth regimes with the predominant processes: Nucleation (I), diffusion-mediated coalescence (II) (*D > 2R*), and adsorption-mediated cluster growth (III) until the percolation threshold (*D = 2R*). Afterwards, movement of grain boundaries leads to a permanent growth of a dominant cluster at the expense of the adjacent clusters (IV). Adapted from reference [133] with permission from the Royal Society of Chemistry, 2013.

In order to understand the generic growth laws, a simplified model system of Au coating on a hard surface (amorphous silicon oxide, SiO*_x_*) was investigated. The sputter deposition process with an effective gold deposition rate *r_eff_* = 0.21 nm/s was continously recorded with a 15 millisecond time resolution [133]. It becomes obvious that for deposition rates under conditions relevant for industrial manufacturing (*r_eff_* > 1 nm/s) a high time resolution is mandatory to observe the early stages of nanocluster growth. As a result of this study, phase transitions and four different stages of growth including their thresholds with sub-monolayer resolution were identified during the first 10 nm of deposited gold. Each stage can be characterized by a predominant surface process and its intrinsic kinetics: nucleation, diffusion, adsorption and grain growth. The quantitative analysis is based on an analytical geometrical model. This novel model allowed simulating, visualizing and unambiguously interpreting gold nanocluster growth kinetics in terms of nanoscopic processes. Thus, morphological real space parameters were extracted, such as cluster size and shape, center-to-center distance, layer porosity and surface coverage (Figure 4), being of prime importance for plasmonics, sensors, and catalysis [46,47,200,201,202,203]. This fundamental approach allows for analyzing complex nanostructures, as we will see below.

**Figure 5:**
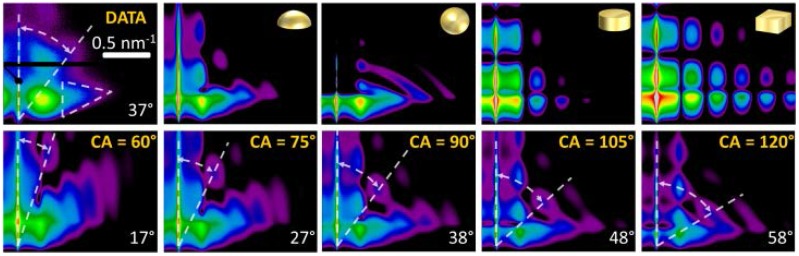
Sputter deposition of Au on amorphous silicon oxide. The upper rows shows the grazing incidence small-angle X-ray scattering (GISAXS) data at δ_Au_ = 6.3 nm (upper left pattern), compared with model-based simulations of expected GISAXS pattern for different cluster geometries (upper row). The geometry of the cluster is depicted in the upper right corner of each pattern. The form factor of the clusters leads to characteristic shapes of the out-of-plane peaks (in horizontal direction) and leads to changes in the intensity and sharpness of height modulations (vertical direction). The curvature of the 2D intensity distribution in the region of the white triangle is indicative of the spheroidal geometry. The lower row depicts the expected GISAXS pattern, obtained by simulations, of spherical clusters with different cluster-to-substrate angle (*CA*), i.e. truncated spheres (lower row). A hemisphere corresponds to *CA* = 90°, which perfectly matches the data. The white number denotes angle between scattering plane and second order height maxima (dashed white lines). Adapted from reference [133] with permission from the Royal Society of Chemistry, 2013.

Furthermore, changes in the nanoparticles’ aspect ratio and the onset of long-range connectivity were deduced during the sputter deposition process. In the nanoscale regime, this traceability is a novel concept and can be extended to analyze sputter deposition on polymeric and functionalized surfaces, where complex nanostructures might occur. The results of this analysis compare very well with the measurement of the optoelectronic properties of ultra-thin gold layers reported in literature [204,205,206]. The analysis of the cluster shape, which is a general and powerful feature of GISAXS, will be outlined in the following, see Figure 5. The GISAXS pattern of physically possible cluster geometries, namely hemisphere, full sphere, cylinder and parallelepiped, were simulated, as outlined above. In detail, at a given fixed distance distribution of the clusters, only the form factor was varied with the additional boundary condition of equal volume for the different cluster shapes. By comparing the simulated GISAXS pattern with the data, it is evident, that the characteristic triangular curvature of the out-of-plane scattering, enhanced by the dashed, white triangle in Figure 5, is clearly related to a spherical geometry. In addition, the intensity and number of height modulations along the vertical (*q_z_*) become more pronounced in planar geometries [99,150]. Furthermore, the exact aspect ratio (being 0.5 for hemisphere) is verified in the second line of Figure 5. The cluster-to-substrate angle (*CA*) denotes the angle of intercept of the envelope of the spherical Au cluster with the substrate surface. This cluster-to-substrate angle was varied by changing the aspect ratio (ratio of height to radius of the Au cluster of the spheroid with constant volume. The characteristic feature in this sequence is the angle between the scattering plane and a diagonal line starting at the horizon and drawn through the second order of the first height peak (Figure 5, white dashed lines). This angle increases almost linearly with cluster-to-substrate angle and thus enables to deduce the wetting behavior of gold on silicon oxide on the nanoscale. In comparison to the simulations, the angle in the measured GISAXS pattern of 37° (between the white lines) yields a cluster-to-substrate angle of *CA* = (90 ± 5)° and allows for proving the hemispherical cluster geometry during growth [133]. This result was further corroborated in literature [179,207] and compares well with the equilibrium contact angle of Au on SiO*_x_* obtained by the Owens, Wendt, Rabel, Kaelble (OWRK)-method [208,209].

The geometrical model correlates in a straightforward manner morphological parameters and technical relevant properties of gold cluster assemblies. Especially, this issue provides the requirements to manufacture defined surface morphologies with tailored properties by self-organization processes. This novel approach was an important prerequisite for further investigations of the influence of different process parameters on the thin metal film morphology such as surface temperature and deposition rates.

**Figure 6:**
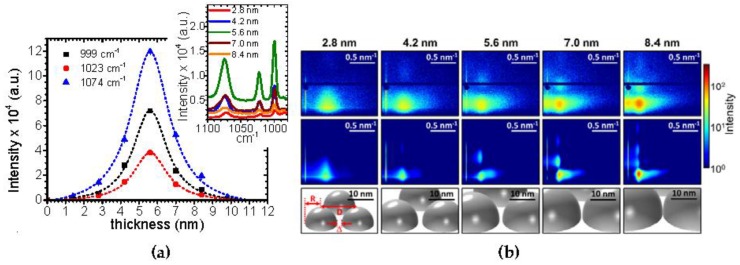
Sputter deposition of Ag on amorphous silicon oxide. (**a**) Surface enhanced Raman scattering (SERS) signals from 10^−7^ mM thiophenol at different deposited silver thicknesses, having different sputter deposited Ag nanostructures on amorphous silicon oxide. The inset shows SERS signal as a function of wavelength for different Ag layer thicknesses. (**b**) Upper row: GISAXS data (upper row) at the different Ag thicknesses indicated above each pattern. Middle row: Model-based simulations (middle row) of the expected GISAXS pattern of the corresponding real-space model (bottom row). The maximum SERS signal corresponds to an effective Ag thickness of δ_Ag_ = 5.6 nm. *D* denotes the center-to-center distance, *R* the cluster radius and Δ the void size. Reproduced with permission from [169]. Copyright AIP Publishing, 2014.

Based on these results, the relationship between SERS activity and silver nanoparticle morphology and structure could be elucidated [169]. The main results are shown in Figure 6. The geometrical model approach was used to analyze the growth kinetics of Ag on SiO*_x_* and to facilitate the interpretation of the morphologies. Furthermore, the model was successfully applied to explain the correlation of the gaps between the nanoparticle and Raman scattering enhancement for supported silver clusters in sensor applications. In particular, control over the extension of the nanoparticle gaps is of vital importance during deposition because these nanogaps determine the interaction between neighboring metallic nanoparticles and thus directly affect the functional properties. Plasmonic properties, for instance, strongly depend on the coupling between the neighboring dipoles, and electronic properties depend exponentially on the width of the tunneling barrier [210]. Based on a theoretical description of surface plasmons between spherical noble metal particles, the enhancement factor for localized surface plasmons is simplified proportionally to the fourth power of the diameter to the interparticle gap ratio [211,212]. Due to the very small interparticle distances between the clusters near percolation, the field enhancement would theoretically amplify the vibrational field of a molecule in the gap (hot spot) by more than 10^10^ times. The results showed an increase of SERS activity with increasing effective film thickness up to a maximum at 5.6 nm, which are a few nanometers lower than the calculated final percolation threshold (Figure 6b). This is due to the fact that the gaps should not be too small that a small molecule like in that case thiophenol still fits in between and reaches the hot spot [208].

**Figure 7:**
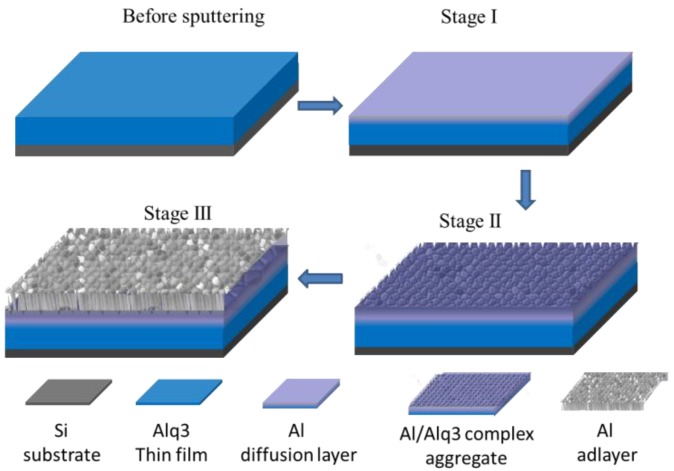
Sputter deposition of Al on Alq3. Three-step growth mechanism similar to Stranski-Krastanov growth for Al, sputter deposited on Alq3. The three stages correspond to the establishment of an enrichment layer with no cluster structures (Stage I), cluster growth on top of the enrichment layer (Stage II), and subsequent columnar growth (Stage III) with increasing Al layer thickness. Reproduced with permission from [168]. Copyright American Chemical Society, 2013.

In a next step, Yu et al. followed the growth of Ag during sputter deposition on an evaporated metal-organic Alq3 layer [213]. Alq3 (tris(8-hydroxyquinolinato)aluminum) is a small molecule containing a complexed Al atom commonly used in organic light emitting diodes [214]. In contrast to Au deposition as outlined above, a different growth mode was found. A Stranski-Krastanov growth is observed, i.e., the surface is first wetted by an Ag layer and upon further growth clusters form on the closed Ag layer. Similar results were found for sputter deposition of Al on the same substrates [168]. A three-step growth mechanism was suggested, where, however, Al is first diffusing into the Alq3 layer and potentially reacts with the complexed aluminum within the first 3 nm of deposition (Figure 7). When this interface is saturated, small nanoparticles grow on top and form nanopillars with ongoing deposition due to minimization of strain.

**Figure 8:**
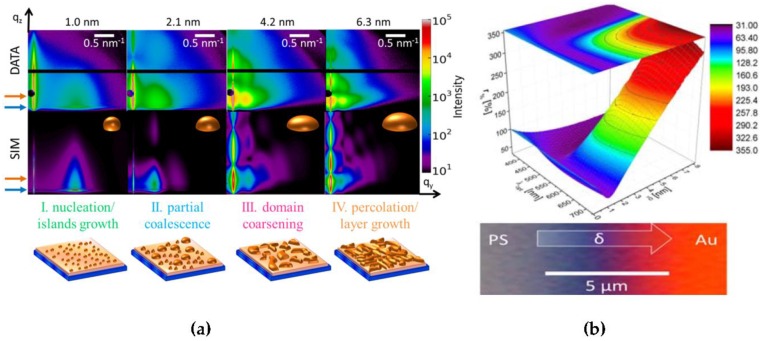
Sputter deposition of Au on PS. (**a**) Upper row: Selected 2D GISAXS patterns illustrate the changes in the GISAXS pattern with increasing effective Au film thicknesses δ_Au_. The critical angles of PS and Au are indicated by the blue and orange arrows, respectively. The beam stop to shadow the specular reflected beam is seen as the black circle, while the intermodule gap is visible as a horizontal black stripe. (*q_y_, q_z_*) denote the reciprocal space coordinate systems. Middle row: model-based simulation of the GISAXS pattern, based on the object shape sketched in the upper right corners. Lower row: Sketch of the cluster growth morphology with ongoing sputter deposition in the four regimes (I–nucleation & islands growth; II–partial coalescence; III–domain coarsening; IV–percolation & layer growth) is indicated (**b**) Change in optical reflectivity *r%* during the deposition process as a function of wavelength $\lambda_{\mathrm{opt}}$ and effective thickness δ_Au_ and optical micro-graph of a corresponding Au gradient, illustrating the change in optical reflectivity of the pristine grey-blue PS film from dark blue color due to the presence of isolated nanoclusters at the interface to bright red color stemming from larger Au aggregates. Reproduced with permission from [144]. Copyright American Chemical Society, 2015.

The abovementioned results present the metal sputter deposition on inorganic substrates and small molecule layers; they proved the principles of the fundamentally different growth modes with different deposited metal and substrate materials and allowed for correlating the nanostructures with its functional properties. On the other hand, it is very attractive to investigate the optical properties of the metal layer during sputter deposition in situ [215] and to correlate them in situ with their detailed nanostructure using GISAXS [216]. Hence, in a next step, real-time monitoring of Au growth morphologies on a PS substrate during sputter deposition using GISAXS and UV-Vis Specular Reflectance Spectroscopy (SRS) was combined in situ. Being very surface sensitive, these techniques facilitate the simultaneous study of thin film morphologies and their optical properties, respectively [217,218]. This allowed in a unique way to correlate the morphological evolution of the nanostructured Au film on PS with the related optical properties, such as antireflective behavior and color changes in the UV-Vis regime during radio frequency (RF) sputter deposition. This fundamental approach using the model system Au on a spin-casted PS thin films allows for tailoring the optoelectronic properties of polymer–metal interfaces [219]. The investigated Au/PS/Si nanocomposite thin films exhibited at different thicknesses significant changes in their visible color and the observed surface and interface morphology. A change in optical reflectivity of the pristine grey-blue PS film was detected ranging from dark blue color due to the presence of isolated nanoclusters at the interface to bright red color from larger Au aggregates during sputter deposition (Figure 8b). In the lower thickness regime of around 1 nm thickness, an anti-reflective behavior was observed, suggesting a promising range for effective antireflective cluster layers in OPV applications to increase their light harvesting capabilities [220,221]. Moreover, four different growth regimes were directly observed: nucleation, isolated island growth, growth of larger aggregates via partial coalescence and continuous layer growth from the course of the key scattering features in the real-time GISAXS experiment as a function of effective gold layer thickness (Figure 8a). Their individual thresholds were identified with subnanometer resolution.

Additionally, a change in cluster aspect ratio (height to radius) was observed and confirmed by simulations, similar to the combinatorial studies (Figure 2) [127,150]. Furthermore, the surface diffusion coefficient of Au on PS at room temperature according to the kinetic freezing model [222] was calculated for the first time based on real-time experiments, comparing well to previous studies [100]. In addition, post-deposition X-ray reflectivity (XRR) confirms the embedding of Au an at the PS–air interface during the deposition, resulting in a 2.3 nm gold enrichment layer with 23% of bulk Au density.

In summary, detailed information on the physico-chemical and electronic parameters as well as the nanostructure is obtained from the presented experiments. This approach is of high relevance for controlling the nanostructure of polymer–metal interfaces down to the sub-monolayer regime for applications in plasmonics, photovoltaics and many other applications. Moreover, it permits a deeper understanding of the growth kinetics of complex metallic nanostructures on polymer substrates, particularly in conjunction with computer simulations of molecular dynamics [159]. According to these findings [159], the structure and morphology of the sputter-deposited layer changes with the sputter rate. Here, the use of one order of magnitude larger sputter rates than in [133] was additionally studied. This leads to sputter rates on the order of 1 nm/s, where one would the ideally use a millisecond time resolution. All these findings help to efficiently control manufacturing of Au cluster films in the multidisciplinary fields ranging from photovoltaic applications, heterogeneous catalysts to semiconductor industry and, in general, in all nano- and surface-related science. From a global perspective, this in turn allows for improved energy usage and saving unique resources as gold, which indeed is of broad appeal, world-wide and ever-growing environmental interest.

### 3.4. Complex Nanostructure Formation Using Nanostructured Polymer Thin Films

One versatile approach to creating complex and hierarchical metal nanostructures is the usage of diblock-copolymer scaffolds, e.g., polystyrene-block-poly(methyl methacrylate) (PS-b-PMMA), as template for vacuum deposition [66]. Due to the molecular incompatibility of the different polymer-blocks, the template is phase separated into regular domains in the order of 10–100 nm, depending on the block length and chemistry. In case of Au sputter deposition on PS containing diblock copolymer thin films, a preferential accumulation of Au at PS domains occurs leading to an imitation of the substrate domain structure by the metal and, hence, to self-assembly of metallic nanostructures [170,66,223]. This selective wetting behavior is primarily attributed to the differences in surface mobility and interaction of the metal adatoms with the PS domains. Based on differences in polymer–metal interaction and interface energy, the intrinsic growth kinetics, respectively wettability and the evolution of the collective optical properties, are very sensitive to variation in the metal as a target material and the composition of the polymeric template [67,100,112,156].

Directional hierarchical nanostructures can be fabricated by exploiting oblique angle sputter deposition and the selective wetting behavior of Au on diblock copolymer thin film. The selectivity of Au on PS domains replicates the diblock-copolymer nanostructure. On the PS domains themselves, Au nanoparticles are present. The oblique angle sputter deposition leads to an inclined growth of the Au nanoparticles with respect to the surface normal [182,224,225]. Thus, one is able to fabricate directional hierarchical nanostructures with optical anisotropy [170].

In the field of colloidal layers, a more complex option is the use of colloidal arrays as a template for sputter deposition. Ultra-thin layer growth of Co on isolated PS colloidal spheres (having a diameter of 100 nm) was investigated. Though rarely visible in SEM images, GISAXS clearly revealed the smooth capping and a continuous wetting of the PS spheres with Co well below the percolation threshold [226]. In the same manner, cadmium-selenide quantum dots are used as templates for gold cluster growth [227]. Here, the quantum dots initially act as nucleation sites for gold growth. In later stages, the gold nanoparticles surrounding the quantum dots undergo a coarsening to form a complete capping layer comprised of gold-dot clusters.

## 4. Outlook

The here presented results provide a first understanding of the deposition stages of the metal sputter deposition process on polymers ranging from homopolymer substrates to complex nanostructured templates, especially for the early stages of growth. In the next step, the combination of sputter deposition with time-resolved GISAXS will explore in situ the nanostructural evolution of gold nanoclusters on polystyrene thin films as a function of the sputter deposition rate. One expects, that this parameter—being crucial for industrial, large scale applications where high-throughput is needed—will influence the metal layer morphology on the polymer thin film [159]. Thus, the impact of different deposition rates on the nucleation regime and the onsets of long-range connectivity during the deposition process can be identified. The effect of a bias voltage on the metal layer morphology [228] may also be investigated in real-time; increasing the energy of the impinging ions alters the roughness of the metal layers and may lead to an increase in the surface defect density of the polymer film which in turn is expected to increase the nucleus density [229,230,231].

In future investigations, even more complex processes and sample systems will be addressed. Biopolymers [232,233,234], such as cellulose and derivates [235], offer a sustainable [236,237] approach for nanostructured thin films [238,239] and nanocomposite thin films [240], paving the way for utilizing superior mechanical properties found in bulk [241,242,243]. Thus, one big challenge is the real-time observation of the sputtering of 2D nanogranular alloys, which offer additional degrees of freedom to tailor the functional properties of polymer–metal interfaces [1,156]. For many electronic, optical, magnetic and other functional properties, nanoalloying exhibit a huge variability, e.g., the particle surface plasmon resonance frequency in plasmonics [2,50,244]. From a fundamental point of view, nanoscale alloy structures can expose marked deviations from bulk phase diagrams [245]. Depending on the size of the nanoobjects, surface and interface energies become important on the nanoscale and below, whereas those contributions can be neglected as the feature size approaches macroscopic dimensions. It will now be very exciting to perform in situ monitoring of nanostructure and crystallite formation as well as electronic and optical properties during phase separation on growing nanostructures. GIXS will decisively aid in establishing a connection between diffusion rate or interface energy and cluster size and shape [177]. A key aspect is the understanding of the complex mechanisms of alloy formation and phase separation on different length scales during selective sputter deposition on organic substrates with an intrinsic nanostructure. The additional degree of freedom is the deposition angle, and advanced nanostructures for metamaterial fabrication, not attainable under normal incidence, can be obtained by glancing incidence deposition [170,225]. All this requires a detailed knowledge of the polymer–metal interface.
